# Differential Subcellular Distribution and Physiological Responses of *Artemisia argyi* to Pb, Cu, and Zn Stress

**DOI:** 10.3390/ijms262311274

**Published:** 2025-11-21

**Authors:** Yu Guo, Xiaoqi Zhang, Tianhong Zhou, Qianqian Yu, Wenfang Hao

**Affiliations:** 1 College of Life Sciences, NorthWest A&F University, Xianyang 712100, China; 2019guoyu@nwafu.edu.cn (Y.G.); zhouth@nwafu.edu.cn (T.Z.); 2024051210@nwafu.edu.cn (Q.Y.); 2 College of Life Sciences, Shaanxi Normal University, Xi’an 710119, China; zhangxiaoqi@snnu.edu.cn

**Keywords:** phytoremediation, *Artemisia argyi*, heavy metals, translocation factor, bioconcentration factor, subcellular distribution

## Abstract

*Artemisia argyi* is recognized as a promising phytoremediation crop due to its high economic value. A 90-day greenhouse pot experiment was conducted to assess the effects of different doses of lead (Pb), copper (Cu), and zinc (Zn) on the growth, heavy metal accumulation, and physiological responses of *A. argyi* cultivated in alkaline soil. The results indicated that exposure to Pb, Cu, or Zn significantly decreased photosynthetic pigment content and induced substantial reactive oxygen species generation, consequently elevating glutathione and malondialdehyde levels. Biomass and heavy metal accumulation data demonstrated that *A. argyi* possesses high tolerance to Pb stress, characterized by low translocation rates and predominant accumulation in roots and cell walls. This retention mechanism supports its potential as a lead-enriching plant while maintaining economic value. In contrast, Zn exhibited the highest translocation rate among the metals tested. Although the proportion of Zn in organelles decreased with increasing stress intensity, its enrichment in organelles remained notably higher than that under Pb or Cu stress. Copper exhibited intermediate mobility, with limited accumulation in organelles, thereby inducing relatively minor cellular damage. These findings elucidate the metal-specific accumulation patterns in *A. argyi* and underscore its potential as a phytoremediation crop for heavy metal-contaminated soils.

## 1. Introduction

Soil is a fundamental resource for human survival, yet it has become increasingly contaminated by heavy metals due to anthropogenic activities. Industrialization, mining, and modern agricultural practices have significantly contributed to the release of these pollutants into the environment. Toxic heavy metals adversely affect soil properties, reduce the availability of essential plant nutrients, and impair plant morphology, structure, and biochemical processes [1,2,3]. Zinc (Zn) and copper (Cu) are essential micronutrients for plant growth and development [4,5]; however they exert toxic effects at elevated concentrations [6,7]. In contrast, non-essential elements such as lead (Pb) and cadmium (Cd) can cause morphological, physiological, and biochemical dysfunctions in plants even at relatively low levels [8].

Heavy metals like Pb and Cd are among the most hazardous pollutants due to their non-essential nature and potential for uptake and accumulation in plants [9,10]. Pb, in particular, is a physiological and neurotoxic agent and is recognized as a naturally occurring human carcinogen [11,12]. It is ranked as the second most hazardous substance after arsenic (As) [13,14]. Widespread contamination by Pb has resulted from industrial and mining activities [15]. In soil, Pb toxicity depends on both its concentration and chemical speciation [16]. Pb binds to inorganic and organic soil components through adsorption, complexation, ion exchange, and precipitation, which reduces its mobility [17,18]. Pb toxicity negatively affects plants throughout their life cycle, with the extent of damage depending on concentration and exposure duration. It inhibits plant growth by interfering with spindle fiber polymerization during mitosis and altering cell wall structure, leading to reduced extensibility and impaired cell elongation [19]. At higher concentrations, Pb disrupts key metabolic processes including the oxygen-evolving complex, organelle integrity, photosystem II connectivity, and the electron transport chain [20,21].

Cu and Zn are essential micronutrients that play critical roles in various physiological and biochemical processes in plants. However, when their concentrations exceed optimal physiological levels, they can induce metabolic disorders and inhibit plant growth and development [22,23]. Cu toxicity primarily manifests through two mechanisms: first, Cu ions disrupt the absorption and transport of other essential nutrients via ion antagonism, leading to deficiencies that further compromise plant health [24]; second, excessive Cu triggers the accumulation of reactive oxygen species (ROS), such as hydroxyl radicals, hydrogen peroxide, and superoxide anions [25]. Similarly, the influence of Zn on plant growth follows a dose-dependent response, wherein low concentrations promote growth, while high concentrations become inhibitory [26,27]. Under Zn stress, key growth and physiological parameters often exhibit an inverted U-shaped dose–response curve. These include biomass, photosynthetic rate, chlorophyll content, and the activities of antioxidant enzymes such as superoxide dismutase (SOD), peroxidase (POD), and glutathione (GSH) [28,29]. Moreover, elevated levels of Cu or Zn can significantly reduce root activity, impair photosynthetic function, and diminish plant growth and yield [30,31].

The subcellular distribution and chemical speciation of heavy metals are key internal mechanisms that plants employ to mitigate metal toxicity [32]. Understanding these patterns is essential for elucidating metal accumulation, translocation, and detoxification strategies, which vary considerably among plant species, cultivars, and ecological types [33]. For instance, in tobacco, Pb is predominantly bound to the cell wall [14]; in *Bidens pilosa* L., Cu is distributed mainly in the cell wall (47%), followed by mitochondria (24%), plastids (22%), and the cytoplasm (6%) [34]; and in *Lactuca sativa* L., soluble components account for 39–44% of Zn storage in roots [35]. Such compartmentalization helps prevent heavy metals from accumulating in metabolically active cellular sites, thereby reducing toxicity [36]. Nevertheless, substantial differences exist in the subcellular distribution and chemical forms of heavy metals across metal types and plant species [37,38]. To date, little is known regarding the subcellular distribution patterns of Pb, Cu, and Zn in *A. argyi*.

Current approaches for managing soil heavy metal contamination primarily encompass physical, chemical, and biological remediation. Among these, phytoremediation has emerged as a sustainable technology developed in recent years for extracting toxic pollutants from the environment [39,40]. Unlike conventional physical and chemical methods, phytoremediation offers advantages such as low cost, minimal disruption to soil and aquatic ecosystems, and no secondary pollution [41,42,43]. In practical applications, two categories of plants are commonly employed: hyperaccumulators and heavy metal accumulators. While the latter exhibit lower accumulation capacity than hyperaccumulators, they typically demonstrate high tolerance to pollutants and produce substantial biomass, encompassing species such as crops, weeds, and woody plants [44,45].

*Artemisia argyi* is a perennial herb in the Asteraceae family, valued for its rich chemical composition, including phenylpropanes, organic acids, flavonoids, and terpenoids, which contributes to its significant economic utility. *A. argyi* has been used for over 3000 years and is widely employed in the pharmaceutical industry as a raw material for moxibustion and medicinal formulations [46]. Its demonstrated antioxidant, moisturizing, and anti-inflammatory properties further support its application in skincare and cosmetic products aimed at slowing the aging process [47]. The capacity of *Artemisia* species to accumulate heavy metals is well documented. For example, *Artemisia* parviflora leaves have been shown to accumulate higher levels of Pb and Zn compared to other medicinal herbs [48], and soil-grown *Artemisia* can grow normally under Cu stress at 60 mg·kg^−1^ [49]. However, although the tolerance of *A. argyi* to Cd stress has been experimentally demonstrated [50], its response to other heavy metals such as Pb, Cu, and Zn remains poorly understood.

*Artemisia argyi* is a traditional Chinese medicinal plant and a heavy metal-tolerant crop with significant economic and practical value. However, research on its potential use in soil remediation has so far been limited to Cd. In this study, we used leaves of *A. argyi* to investigate the differential regulatory mechanisms under Pb, Cu, and Zn stress at varying concentrations. We measured multiple physiological indicators, analyzed metal accumulation and translocation patterns, and determined subcellular distribution. This study aimed to clarify the tolerance of *A. argyi* to Pb, Cu, and Zn and to identify appropriate scenarios for its phytoremediation application. We hope to identify the reasons why *A. argyi* can serve as an enrichment plant for Pb and a hyperaccumulation plant for Zn remediation by studying the differences in enrichment rates, migration rates, and subcellular enrichment mechanisms of different heavy metals, and provide theoretical guidance for Cu remediation.

## 2. Results

### 2.1. Growth Changes

**Figure 1:**
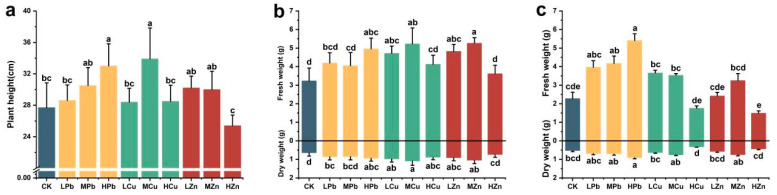
Effects of Pb, Cu, and Zn stress on growth parameters of *A. argyi*: (**a**) plant height; (**b**) fresh and dry weight of the aboveground part; (**c**) fresh and dry weight of the belowground part. Lowercase letters above bars indicate significant differences among treatments (*p* < 0.05).

Exposure to low concentrations of Pb, Cu, and Zn individually promoted the growth of *A. argyi* compared to the control group (Figure 1). Plant height, fresh weight, dry weight, and dry mass percentage across different plant parts were analyzed. The results showed that plant height under low and medium concentrations of all three metals exceeded that of the control. Under Pb treatment, plant height increased with rising concentration, showing a significant increase of 19.13% under HPb treatment relative to the control. In contrast, under Cu treatment, plant height initially increased and then decreased with concentration, reaching a significant peak increase of 22.38% under MCu conditions. Under Zn treatment, plant height consistently declined with increasing concentration, decreasing by 8.30% under HZn treatment compared to the control (Figure 1a). Overall, plant height exhibited a “low-promotion, high-inhibition” response pattern.

Aboveground biomass of *A. argyi* increased under all metal treatments compared to the control. Fresh weight of the aboveground parts was significantly higher under HPb, LCu, MCu, LZn, and MZn treatments. Fresh weight of belowground parts also increased significantly under Pb treatment. The most notable increase in aboveground biomass occurred under Cu treatment, with a significant increase of 61.81% under MCu relative to the control. Under both Cu and Zn treatments, aboveground biomass showed an initial increase followed by a decline as metal concentrations rose. Meanwhile, the drying rate of aboveground tissues was higher under HCu treatment compared to the control. Belowground biomass increased significantly under Pb stress, with the fresh weight of belowground parts increasing by 137.36% under HPb treatment, indicating substantially enhanced tolerance to Pb (Figure 1b,c).

### 2.2. Oxidative Stress-Related Indicators

**Figure 2:**
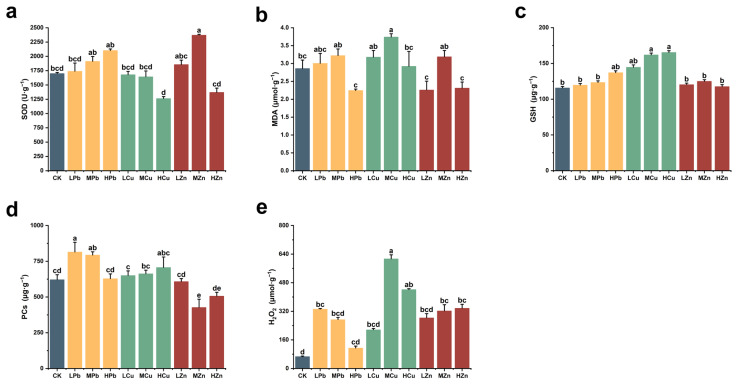
Effects of Pb, Cu, and Zn stress on oxidative stress indicators in *A. argyi* plants: (**a**) Superoxide dismutase (SOD) activity; (**b**) Malondialdehyde (MDA) content; (**c**) glutathione (GSH) content; (**d**) Phytochelatins (PCs) content; (**e**) H_2_O_2_ content. Lowercase letters above bars indicate significant differences among treatments (*p* < 0.05).

To assess the impact of Pb, Cu, and Zn stress on oxidative stress-related indicators in *A. argyi* leaves, we measured the activity of SOD and the contents of MDA, GSH, PCs, and H_2_O_2_ (Figure 2). Under Pb stress, SOD activity increased gradually with rising Pb concentration, reaching its highest level under HPb treatment, which was 23.74% higher than that of the control. In contrast, under HCu and HZn treatments, SOD activity decreased by 25.66% and 19.35%, respectively, compared to the control (Figure 2a). MDA content decreased by 21.41% under HPb stress and by 19.13% under HZn stress relative to the control. Under Cu stress, MDA content was consistently higher than that of the control and exhibited an initial increase followed by a decrease with increasing Cu concentration. Under MCu stress, MDA content increased significantly by 30.86% compared to the control (Figure 2b). GSH content under Pb, Cu, and Zn stress was elevated compared to the control. Under both Pb and Cu stress, GSH content rose continuously with increasing stress intensity, showing increases of 18.42% under HPb and 42.87% under HCu stress. The change in PCs content under Cu stress was positively correlated with that of GSH. PCs content increased by 31.11% under low-Pb (LPb) and by 13.58% under HCu stress. Under Zn stress, GSH content showed an initial increase followed by a decrease, rising by 7.96% under MZn stress, while PCs decreased by 31.43% under the same conditions. H_2_O_2_ content, an important indicator of oxidative damage, increased significantly under all metal stresses compared to the control. Under Pb stress, H_2_O_2_ content gradually decreased with increasing stress level, though it remained 400.97% higher under LPb stress than in the control. The most pronounced increase in H_2_O_2_ occurred under MCu stress, which showed an 825.49% rise compared to the control. Under Zn stress, H_2_O_2_ content increased progressively with stress intensity, reaching 508.39% higher than the control under HZn treatment (Figure 2e). These results indicate that Pb, Cu, and Zn stress elevate ROS levels in *A. argyi*, leading to increased accumulation of antioxidant substances. However, high concentrations of these metals may disrupt normal antioxidant responses.

### 2.3. Photosynthetic Pigments

**Figure 3:**
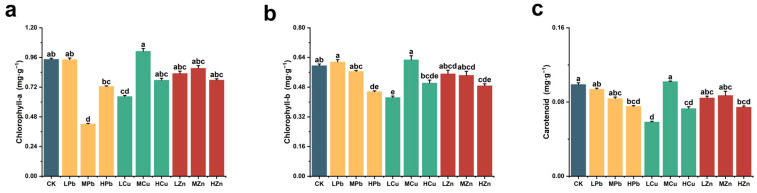
Photosynthetic pigment content in *A. argyi* under Pb, Cu, and Zn treatments: (**a**) chlorophyll a content; (**b**) chlorophyll b content; (**c**) carotenoid content. Lowercase letters above bars indicate significant differences among treatments (*p* < 0.05).

Figure 3 illustrates the effects of Pb, Cu, and Zn stress on photosynthetic pigment content in *A. argyi*. Compared to the control, HPb stress reduced chlorophyll a, chlorophyll b, and carotenoid content by 23.14%, 23.48%, and 23.82%, respectively. Under Cu stress, photosynthetic pigment content showed an initial increase followed by a decrease with rising stress intensity. At LCu stress, chlorophyll a, chlorophyll b, and carotenoid content decreased by 31.71%, 28.80%, and 40.89%, respectively. Under Zn stress, HZn treatment had the most pronounced inhibitory effect, reducing chlorophyll a, chlorophyll b, and carotenoid content by 17.68%, 18.16%, and 24.77%, respectively. These results indicate that high concentrations of Pb, Cu, and Zn stress suppress photosynthetic pigment production in *A. argyi*.

### 2.4. Osmotic Regulation Substances

**Figure 4:**
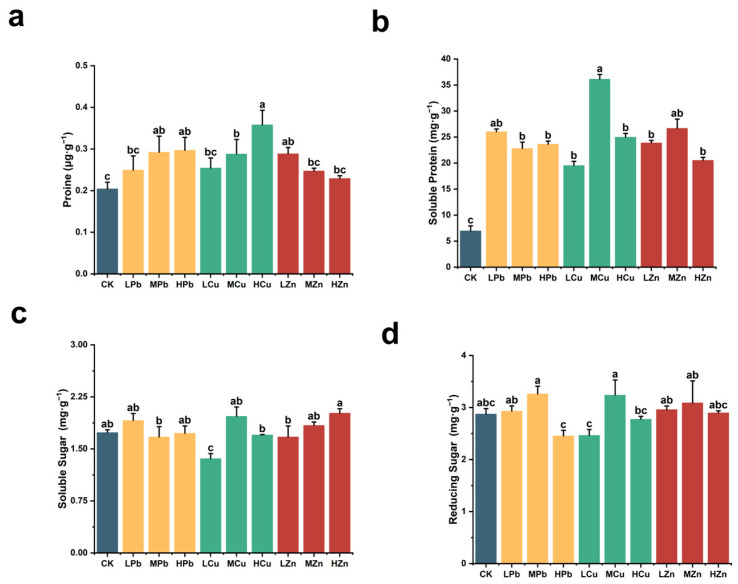
Effects of Pb, Cu, and Zn stress on osmotic regulators in *A. argyi* plants: (**a**) proline content; (**b**) soluble protein content; (**c**) soluble sugar content; (**d**) reducing sugar content. Lowercase letters above bars indicate significant differences among treatment (*p* < 0.05).

To evaluate the effects of Pb, Cu, and Zn stress on osmoregulatory substances in *A. argyi* leaves, we measured the contents of proline (Pro), soluble protein, soluble sugar, and reducing sugar under each treatment condition (Figure 4). Proline content, a key indicator of abiotic stress response, increased with rising stress intensity under Pb and Cu treatments, showing significant increases of 45.42% under HPb and 75.39% under HCu compared to the control. In contrast, under Zn stress, proline content decreased progressively with increasing concentration, though it remained above control levels (Figure 4a). Soluble protein content increased significantly under all metal stresses compared to the control. The most notable increase occurred under MCu stress, with a rise of 419.22%. Under LPb and MZn stress, soluble protein content increased by 273.92% and 283.17%, respectively (Figure 4b). Both soluble sugar and reducing sugar content increased under LPb, MCu, MZn, and HZn treatments relative to the control. Under MPb stress, reducing sugar content increased by 13.46%, while soluble sugar content showed a slight but non-significant decrease of 3.61%. Reducing sugar content under Pb, Cu, and Zn stress exhibited an initial increase followed by a decline with increasing metal concentration, rising by 12.69% under MCu and 7.47% under MZn stress (Figure 4c,d). These results indicate that low concentrations of Pb, Cu, and Zn enhanced osmotic adjustment capacity and increased the levels of protective osmolytes in *A. argyi* leaves. Under high concentrations, however, the content of these protective substances tended to decrease, consistent with the typical “low-promotion, high-inhibition” response pattern in plants.

### 2.5. Pb, Cu, Zn Contents in A. argyi

**Figure 5:**
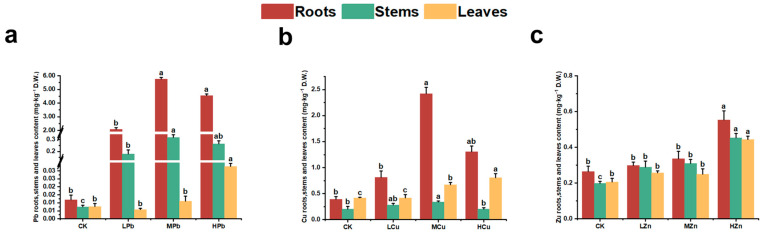
Content of Pb, Cu, and Zn in different tissues of *A. argyi*: (**a**) Pb content in plant tissues; (**b**) Cu content in plant tissues; (**c**) Zn content in plant tissues. Lowercase letters above bars indicate significant differences among treatments (*p* < 0.05).

**Figure 6:**
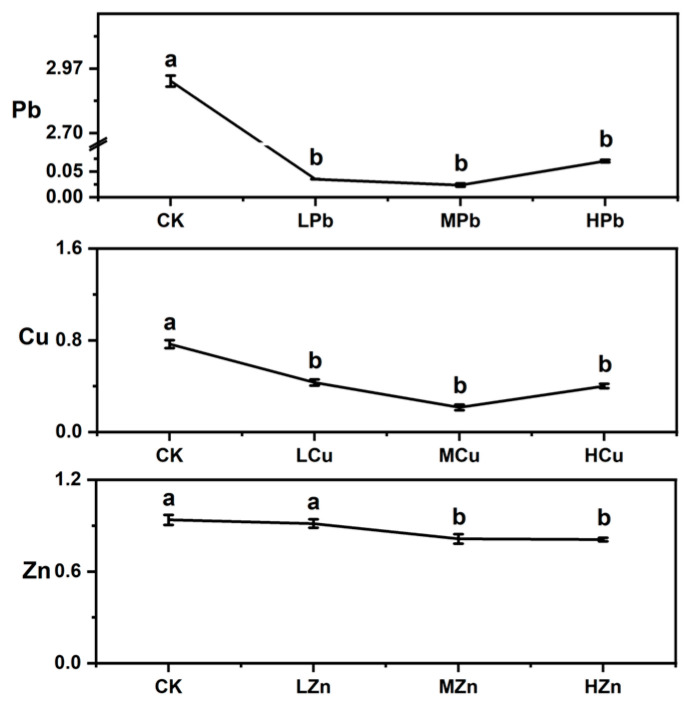
Transfer Factor (TF) of Pb, Cu, and Zn in *A. argyi*. Lowercase letters above bars indicate significant differences among treatments (*p* < 0.05).

The distribution of Pb, Cu, and Zn in roots, stems, and leaves of *A. argyi* under metal stress is shown in Figure 5. Under Pb stress, Pb was predominantly accumulated in the roots. The translocation factor (TF) showed an initial decrease followed by an increase with rising stress intensity. Under MPb stress, Pb content in roots, stems, and leaves was 5.759, 0.294, and 0.011 mg·kg^−1^ DW, respectively, with a TF of 0.024. As stress levels increased, Pb content in leaves gradually rose, reaching 0.033 mg·kg−1 DW under HPb stress—a 194.77% increase relative to MPb stress (Figure 5a and Figure 6). Under Cu stress, Cu was mainly accumulated in aboveground tissues, particularly in leaves. Under LCu stress, TF was lowest (0.432), with Cu contents in roots, stems, and leaves measuring 0.813, 0.278, and 0.418 mg·kg^−1^ DW, respectively (Figure 5b and Figure 6). Under Zn stress, Zn distribution was more uniform across roots, stems, and leaves. The highest TF (0.913) occurred under LZn stress, with Zn contents of 0.298, 0.289, and 0.257 mg·kg^−1^ DW in roots, stems, and leaves, respectively (Figure 5c and Figure 6). Analysis of tissue metal content across treatments indicated that TF values were highest under Zn stress, followed by Cu and Pb. Overall, under Pb, Cu, and Zn stress, metal elements were predominantly enriched in root tissues.

### 2.6. Subcellular Distribution of Pb, Cu, Zn

**Figure 7:**
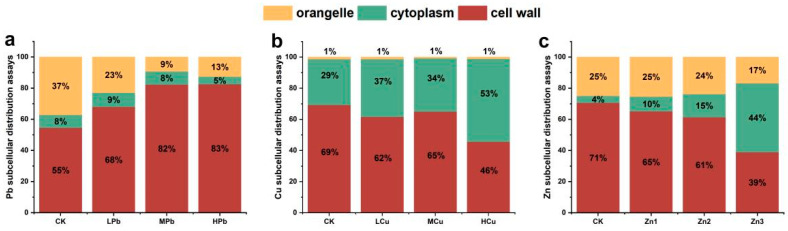
Subcellular distribution of Pb, Cu, and Zn in different tissues: (**a**) Pb distribution; (**b**) Cu distribution; (**c**) Zn distribution.

Given the distinct patterns of intracellular enrichment of heavy metals in *A*. *argyi* under Pb, Cu, and Zn stress, their subcellular distribution in leaves was analyzed across treatment groups (Figure 7). The results demonstrated that under Pb stress, Pb was primarily enriched in the cell wall, followed by organelles and soluble components. As the stress intensity increased, the proportion of Pb in the cell wall gradually rose. Compared to the MPb treatment, the HPb treatment showed an increased proportion of Pb in the cell wall, while the proportion in soluble components decreased from 8% to 5% (Figure 7a). Under Cu stress, the proportion of Cu in the cell wall decreased significantly under HCu treatment, whereas the proportion in soluble components increased markedly from 37% to 53%. The proportion of Cu in organelles remained a low level (Figure 7b). Under Zn stress, the proportions of Zn in the cell wall and organelles decreased continuously with increasing stress, while the proportion in soluble components consistently increased from 10% to 44% (Figure 7c). In summary, under Pb, Cu, and Zn stress, the heavy metals were mainly enriched in the cell wall. However, with increasing metal concentrations, a greater proportion of Cu and Zn was redistributed to soluble cellular components.

### 2.7. Principal Components Analysis (PCA) and Correlation Analysis of Growth and Physiological Indicators in A. argyi

**Figure 8:**
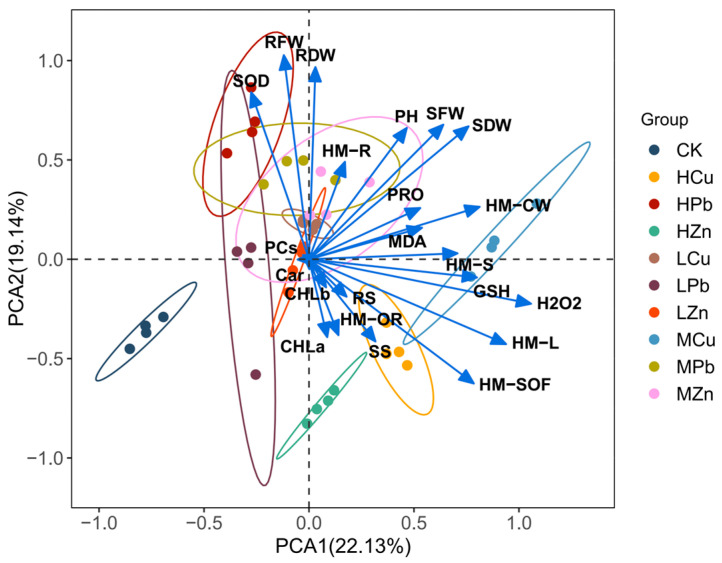
PCA of physiological indicators in *A. argyi* under different heavy metal treatments.

To further examine the relationships among growth and physiological indicators in *A. argyi* under heavy metal stress, principal component analysis (PCA) was conducted on 27 measured parameters. Two principal components were extracted, accounting for 22.13% (PC1) and 19.14% (PC2) of the total variance. The control group exhibited clear separation from the metal-treated groups, with the HZn treatment showing the most distinct profile. Overall, Pb, Cu, and Zn stress significantly altered the physiological and biochemical indicators of *A. argyi*, and differences were also evident among concentration levels within each metal treatment (Figure 8).

**Figure 9:**
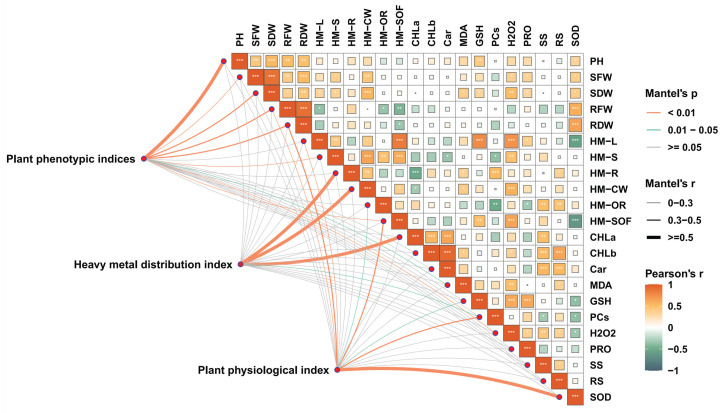
Correlation network among phenotypic, heavy metal distribution, and physiological indicators. Plant phenotypic indices (plant height, shoot biomass, root biomass), heavy metal content (in shoot, root, and subcellular components), and physiological indices (photosynthetic pigments, osmoregulatory substances, oxidative markers) were analyzed using the Mantel test. Edge width corresponds to Mantel’s r statistic, and color represents statistical significance based on 999 permutations. The color gradient indicates Pearson’s correlation coefficient (*p* < 0.05, *p* < 0.01, *p* < 0.001). Abbreviations: PH, plant height; SFW, shoot fresh weight; SDW, shoot dry weight; RFW, root fresh weight; RDW, root dry weight; HM-L, heavy metal content in leaves; HM-S, heavy metal content in stem; HM-R, heavy metal content in root; HM-CW, heavy metal content in cell wall; HM-OR, heavy metal content in organelles; HM-SOF, heavy metal content in soluble fraction; CHLa, chlorophyll a content; CHLb, chlorophyll b content; Car, carotenoid content; MDA, malondialdehyde content; GSH, glutathione content; PCs, phytochelatin content; H_2_O_2_, hydrogen peroxide content; PRO, proline content; SS, soluble sugar content; RS, reducing sugar content; SOD, superoxide dismutase activity.

Correlation analysis revealed that under Pb, Cu, and Zn stress, the heavy metal content in aboveground tissues was positively correlated with H_2_O_2_ content, GSH content, and metal content in the cytoplasm, but negatively correlated with SOD activity, carotenoid content, and photosynthetic pigment content. In contrast, heavy metal content in belowground tissues was positively correlated with PCs content, reducing sugar content, and cell wall-bound metal content, while negatively correlated with chlorophyll a content, cytoplasmic metal content, and soluble fraction metal content (Figure 9).

## 3. Discussion

### 3.1. Effects of Pb, Cu, Zn Stress on Aboveground Belowground Parts of A. argyi

Pb is a non-essential element for plants and animals and is recognized as the second most toxic metal after As due to its low mobility and high toxicity. Cu and Zn also inhibit plant growth at elevated concentrations. Plant morphology and biomass yield serve as direct indicators of heavy metal tolerance [51]. Previous studies have shown that high concentrations of heavy metals in soil can induce multiple toxic effects in plants, including interference with nutrient uptake, reduced water use efficiency, and induction of ROS bursts. These stresses ultimately inhibit photosynthesis and can trigger cell death, leading to substantial reductions in crop yield and quality [52]. In this study, the biomass of *A. argyi* increased significantly under Pb and Cu stress, indicating considerable tolerance to these metals. Under Cu stress, biomass exhibited a “low-promotion, high-inhibition” pattern [53,54]. Low metal concentrations may act as cofactors for enzymes involved in metabolic processes, stimulating the production of stress response substances such as PCs. In contrast, high concentrations disrupt enzyme activity, impair photosynthesis and respiration, and lead to ROS accumulation and inhibitory effects. These findings align with those reported by Srivastava et al. [55] and Souza et al. [56]. Under Zn stress, plant height exhibited a consistent decrease. However, aboveground biomass increased under both LZn and MZn treatments, indicating that plant growth was likely sustained through enhanced tillering or stem thickening rather than vertical extension. Under HZn stress, biomass declined, indicating growth inhibition at this concentration.

Plants in heavily polluted environments employ two primary strategies to minimize damage to aboveground tissues [57]. First, since most absorbed heavy metals accumulate in roots, plants often enhance root activity to adapt to stress and restrict upward translocation. Second, due to the heterogeneous distribution of heavy metals in soil, contamination can disrupt root cell structure and inhibit growth. In response, roots may exhibit an avoidance mechanism by preferentially growing toward less contaminated zones to reduce metal-induced damage [58]. In this study, both biomass and dry weight of the *A. argyi* root system increased significantly under Pb stress compared to the control, indicating that Pb stress stimulates root growth in this species. The low mobility of Pb likely allows the plant to sequester the metal in root tissues, thereby reducing damage to aerial parts and supporting aboveground dry matter accumulation. These results are consistent with findings reported by Murat [59] and Khan et al. [60]. The effects of Zn and Cu stress on the root system of *A. argyi* were concentration-dependent. Treatment with 100 mg·kg^−1^ Cu and 300 mg·kg^−1^ Zn promoted root growth, whereas higher concentrations were inhibitory. This pattern aligns with observations by Daniele [61], who reported biomass reduction in *Hymenaea courbaril* L. under high Cu stress. Excessive Cu and Zn can inhibit cell elongation and division [62,63], disrupt photosynthesis and respiration, and impair water balance. The resulting decline in carbon assimilation compromises plant growth and developmental stability, ultimately leading to reduced biomass [64].

This indicates that Pb, Cu, and Zn exert distinct effects on the growth of *A. argyi*. All three metals can promote plant growth at low concentrations. However, under high concentrations of Cu and Zn stress, the aboveground biomass of *A. argyi* tends to decrease, and root growth is significantly inhibited. In contrast, the effect of Pb stress on *A. argyi* growth is relatively moderate, and its root system exhibits enhanced growth, likely attributable to an avoidance mechanism.

### 3.2. Effects of Pb, Cu, Zn Stress on Oxidative Stress-Related Indicators in A. argyi

Biotic and abiotic stresses can induce oxidative stress and lipid peroxidation in plants [65]. Toxic levels of Pb, Cu, and Zn promote excessive production of ROS such as O_2_^−^ and H_2_O_2_, resulting in oxidative damage to biological components including nucleic acids and proteins [66,67]. The significant increase in H_2_O_2_ content observed in this study under moderate concentration stress is corroborated by the rise in MDA, a terminal product of ROS-induced lipid peroxidation [68]. To counteract heavy metal-induced oxidative stress, plants synthesize various enzymatic and non-enzymatic antioxidants, including proline, ascorbic acid, GSH, and SOD [69]. In this experiment, increased ROS levels were accompanied by significant raised in SOD activity, proline and GSH content, indicating that *A. argyi* activates antioxidant and osmoregulatory mechanisms to alleviate metal toxicity. A decline in SOD and proline under certain conditions may reflect impaired stress resistance or irreversible cellular damage [70]. High concentrations of Cu and Zn caused more pronounced disruption to the antioxidant system of *A. argyi* compared to Pb.

Plants employ two principal metal detoxification mechanisms: the transport of active metal ions into vacuoles and the formation of metal complexes via chelation with metal-binding peptides [71]. Low-molecular-weight metal chelators are recognized as fundamental components of plant metal detoxification. Metal chelates such as PCs, amino acids, MTs, and organic acids play crucial roles in regulating metal toxicity [72]. Among these, GSH serves not only as a key cellular antioxidant but also as a precursor for the synthesis of heavy metal-chelating peptides under metal stress. Studies have reported that under Pb stress, GSH content in the roots of *Triticum aestivum* L. increases significantly, supporting both PC biosynthesis and antioxidant activity [73], which aligns with the findings of this study. Excessive Cu accumulation can induce PC formation and promote the synthesis of PC–Cu complexes in vitro; however, PC-deficient *Arabidopsis* mutants did not show increased sensitivity to Cu stress [74]. This suggests that PCs may not play a primary role in Cu tolerance, that PC–Cu complexes may be transient or partially sequestered in vacuoles, and that other detoxification mechanisms exist for Cu in plants [75]. Similarly, PC levels in *Brassica juncea* L. did not increase zinc accumulation [76], and the absence of Zn-induced PC synthesis in *Thlasci caerulescens* [77] and the absence of Zn-induced PC synthesis in Thlasci caerulescens. These reports are consistent with the lack of a significant increase in PCs under Cu and Zn stress observed here. In contrast, the synthesis of PC–Pb complexes is a key mechanism for Pb detoxification in plants, with PC content closely correlated to Pb accumulation [78]. Under Pb stress, PC content in *A. argyi* increased significantly, supporting its role in Pb tolerance.

### 3.3. Effects of Pb, Cu, Zn Stress on Photosynthetic Pigments in A. argyi

In this experiment, the contents of chlorophyll a, chlorophyll b, and carotenoids decreased significantly with increasing Pb concentration. As a non-essential element, Pb can damage chloroplast ultrastructure and protein complexes, while inhibiting key chlorophyll synthesis enzymes such as δ-aminolevulinic acid dehydratase and protochlorophyllide oxidoreductase [79]. This inhibition negatively affects both chlorophyll content and leaf gas exchange properties [80,81]. Furthermore, Pb stress can induce ROS imbalance, and excess ROS may oxidize chlorophyll molecules, leading to direct degradation of photosynthetic pigments [82,83]. Romanowska et al. [84] reported that under lead stress, mitochondrial respiration (dark respiration) in plants is more severely impaired, whereas photorespiration remains relatively less affected. The maintenance of photorespiration aids in mitigating photoinhibition and oxidative damage, whereas the upregulation of mitochondrial respiration indicates its role as a primary target of lead toxicity. Such metabolic flexibility enhances plant survival in lead-contaminated environments, though it may also result in divergent changes in photosynthetic pigment content and plant biomass. Further investigation integrating molecular markers (e.g., expression of respiration-related genes) with metabolomic approaches is required to elucidate the underlying mechanisms.

Cu and Zn act as structural components in mitochondria, chloroplasts, and certain enzymes. For example, plastocyanin, a Cu-containing protein, participates in electron transfer between photosystem II and photosystem I during the photochemical phase of photosynthesis [85,86]. However, excessive accumulation of Cu and Zn in plant tissues leads to competition with other essential metal ions (e.g., Fe, Ni), displacing metallic cofactors in enzymes. This disruption impairs photosynthetic function, reduces carbon fixation and energy production, and ultimately inhibits plant growth and development [87]. These mechanisms are consistent with the experimental results observed under treatment with 200 mg·kg^−1^ Cu and 600 mg·kg^−1^ Zn.

Changes in carotenoid content generally serve as an indicator of plant developmental stages and responses to environmental stressors, including light, temperature, and drought [88,89]. In the present study, carotenoid content declined following exposure to Pb, Cu, and Zn treatments. This decline may be attributed to high stress intensity; for example, Cu stress has been shown to reduce carotenoids in *Chrysanthemum indicum* L. and *Tagetes erecta* L. [90], and Pb stress can significantly decrease carotenoids in *Brassica juncea* L. [91]. Alternatively, during the maturation of some plants (e.g., *Citrus reticulata* ‘Unshiu’), carotenoid content exhibits an initial increase followed by a decrease [92].

### 3.4. Effects of Pb, Cu, and Zn Stress on the Content of Osmoregulatory Substances in A. argyi

Heavy metal stress can reduce plant cell wall plasticity and induce stomatal closure [93], leading to decreased transpiration. Under these conditions, plants maintain cellular osmotic balance by synthesizing osmolytes such as proline, soluble sugars (e.g., sucrose and trehalose), and soluble proteins (e.g., heat shock proteins), while activating sugar metabolic pathways including glycolysis and the pentose phosphate pathway [94]. These responses help regulate cellular osmotic potential, provide energy, and maintain intracellular water balance. However, high concentrations of heavy metals can reduce the content of sugars, amino acids, and other osmoregulatory substances. In this study, low concentrations of Pb, Cu, and Zn enhanced osmotic regulation and increased the levels of protective osmolytes in leaves, whereas high concentrations led to a decrease in these substances. This pattern aligns with the typical “low-promotion, high-inhibition” response mechanism in plants. Furthermore, under certain treatments, the content of soluble sugars and proline in *A. argyi* increased continuously, which may be attributed to heavy metal stress generating substantial ROS, with soluble sugars participating in ROS-related synthesis and decomposition processes [95]. For example, application of Cu^2+^ and Zn^2+^ has been shown to increase soluble sugar and protein content in *Cladophora* [96].

### 3.5. Accumulation of Pb, Cu, and Zn in A. argyi Plants

In this study, *A. argyi* exhibited strong accumulation capacity for Pb, Cu, and Zn. Metal accumulation generally followed the order: roots > stems and leaves, though Cu distribution in aerial parts showed a distinct pattern of leaves > stems. Under Zn stress, the TF of Zn in *A. argyi* approached 1, indicating efficient upward translocation, which may be attributed to the physiological roles of Cu and Zn in photosynthetic processes [97,98]. However, as Zn and Cu stress levels increased, more Zn was retained in roots, while root enrichment of Cu increased initially and then decreased, suggesting the activation of self-protection mechanisms that limit metal translocation to aerial tissues [37]. This finding aligns with reports by Marco et al. [99], who observed reduced TF values in *Senna multijuga* and *Erythrina crista-galli* under Cu-contaminated soil conditions. Compared to Cu and Zn, Pb shows lower mobility and higher toxicity in plants and is primarily enriched in root systems, consistent with patterns observed in *Nerium oleander* L. and *Brassica juncea* [100]. Finster et al. demonstrated that roots consistently accumulate more Pb than other plant parts, including fruits [101]. The limited translocation of Pb from roots to shoots may result from compartmentalization within cellular structures such as vacuoles, a key defense mechanism against heavy metal stress in plants [102]. In this study, under 1000 mg·kg^−1^ Pb treatment, Pb content in roots began to decrease, while a significant increase occurred in leaves. This suggests that the 1000 mg·kg^−1^ treatment may have exceeded the root storage capacity of *A. argyi*, leading to enhanced translocation to aerial parts and potentially more severe phytotoxicity.

### 3.6. Accumulation of Pb, Cu, and Zn in the Subcellular Components of A. argyi

The subcellular distribution of metal elements directly reflects their accumulation and partitioning within plants and is closely associated with plant adaptability and tolerance under heavy metal stress [103]. Plants can alleviate metal toxicity by sequestering heavy metals in less metabolically active compartments such as cell walls and vacuoles. The plant cell wall, which constitutes the primary barrier protecting the protoplast from metal toxicity, consists mainly of polysaccharides (including cellulose, hemicellulose, and pectin) and proteins. It provides negatively charged binding sites that can immobilize metal ions and restrict their transmembrane transport [104]. This study showed that under Pb, Cu, and Zn treatment, the majority of these metals were retained in the cell wall fraction of *A. argyi*, with Pb exhibiting the highest retention ratio. This indicates that the cell wall helps reduce heavy metal toxicity by immobilizing Pb, Cu, and Zn and limiting their movement across membranes, thereby supporting cellular metabolic function and structural integrity. Vacuoles can accumulate excess heavy metals and provide an additional mechanism for toxicity mitigation. When the binding capacity of the cell wall is exceeded, metal ions may be transported into vacuoles, which represent a major component of the soluble fraction [105]. Under Cu and Zn treatment, the enrichment ratio in the cell wall decreased with increasing stress levels, while the proportion in the soluble fraction increased. At the same time, heavy metal content in organelles remained low, helping maintain a relatively stable cytoplasmic environment in *A. argyi* and supplying steady levels of Cu and Zn for normal growth while preventing excessive cytoplasmic accumulation. Furthermore, the subcellular distribution of heavy metals helps explain their translocation within the plant; greater distribution in soluble fractions may be associated with enhanced translocation from roots to aerial parts [106].

In this study, the proportion of Cu and Zn in the soluble fraction was significantly higher than that of Pb, facilitating the translocation of Cu and Zn ions from roots to shoots. Under 1000 mg·kg^−1^ Pb treatment, the proportion of Pb in the cell wall remained largely unchanged, while Pb content within organelles began to increase. This suggests that the protective barrier function of the cell wall against Pb reached its threshold, allowing Pb to enter organelles, disrupt cellular homeostasis, and impair normal cell function. In summary, the cell wall and soluble fractions serve as the primary storage sites for Pb, Cu, and Zn in *A. argyi* cells. The higher translocation rate of Cu and Zn compared to Pb, combined with the predominant enrichment of Pb in the cell wall, indicates that *A. argyi* exhibits greater tolerance to Pb and is less susceptible to Pb toxicity. The elevated biomass and high root enrichment ratio identify *A. argyi* as a lead-accumulating plant. Its TF approaching 1 suggests potential as a Zn hyperaccumulator. Although the TF under Cu stress exceeded that under Pb stress, it remained a low level, indicating limited copper translocation. Future research may explore the application of exogenous amendments to enhance Cu transport and mitigate its phytotoxicity in *A. argyi*.

## 4. Materials and Methods

The concentrations of heavy metals used in this experiment were selected in accordance with “Soil Environmental Quality—Risk Control Standard for Soil Contamination of Agricultural Land” [107].

### 4.1. Experimental Materials and Potted Plant Experiments

Soil samples were collected from the top layer (0–20 cm depth) of an experimental field at Northwest A&F University (34°17′ N, 108°04′ E). The soil was classified as dark loess (Cambisol) with the following physicochemical properties: organic matter, 7.69 g·kg^−1^; total nitrogen (N), 0.57 g·kg^−1^; total phosphorus (P), 0.66 g·kg^−1^; total potassium (K), 17.2 g·kg^−1^; lead (Pb), 18.405 mg·kg^−1^; copper (Cu), 30.420 mg·kg^−1^; zinc (Zn), 64.948 mg·kg^−1^; and pH 8.1.

Rhizomes of *A. argyi* were collected from Qichun County, Hubei Province, and authenticated as *A. argyi* by Hubei Duanyang Qiai Technology Co., Ltd. (Huanggang, China).

A completely randomized design was employed with ten treatment groups: CK (no addition of Pb, Cu, or Zn); Pb applied as lead acetate at LPb (170.0 mg·kg^−1^), MPb (500.0 mg·kg^−1^), and HPb (1000.0 mg·kg^−1^); Cu applied as copper sulfate pentahydrate at LCu (50.0 mg·kg^−1^), MCu (100.0 mg·kg^−1^), and HCu (200.0 mg·kg^−1^); Zn applied as zinc sulfate heptahydrate at LZn (150.0 mg·kg^−1^), MZn (300.0 mg·kg^−1^), and HZn (600.0 mg·kg^−1^). The compounds were thoroughly mixed with 1.0 kg of soil before placement in plastic pots (10 cm in diameter and 12.2 cm in height). Each treatment included five pots. No additional fertilizers were applied. The soil was equilibrated for two weeks while maintaining its maximum water-holding capacity. Uniform, plump, and disease-free rhizomes were selected and cut into 5 cm-long segments, each containing a single stem and weighing 1.5 g, for planting. Plants were planted on 4 January 2024, with one plant per pot, and watered with 50 mL every two days. The pots were placed in a greenhouse for 90 days under average day/night temperatures of 27/16 °C. Standard cultivation practices were followed. Swap the positions of potted plants inside and outside every week to eliminate the influence of location. Treated plants were sampled after 90 days and immediately preserved for further analysis.

### 4.2. Growth Index Measurement

After 90 days of exposure to heavy metal stress, the aboveground height and the fresh weight of roots, stems, and leaves were measured for each plant. Harvested plants were thoroughly rinsed with distilled water and 20 mM Na_2_EDTA. Each plant was divided into two parts: one for analysis of growth parameters and subcellular distribution (The distribution of elements in cell walls, organelles, and soluble components), and the other for determination of tissue heavy metal concentration. The latter portion was flash-frozen in liquid nitrogen and stored at −80 °C. Samples of leaf, stem, and root tissues were dried in an oven at 105 °C for 30 min, then at 80 °C for 72 h, and dry weight was measured using an electronic balance.

### 4.3. Calculation of Pb, Cu, and Zn Accumulation, TF

Dried roots, stems and leaves were ground, and 0.5 g of each sample was digested with 5 mL nitric acid using a microwave digestion system (Milestone MA165-001 Multiprep-41 FC2, Milestone S.r.l., Sorisole, Italy). The digest was concentrated to 1–2 mL by heating at 130 °C, cooled to room temperature, and diluted to 25 mL with deionized water. Heavy metal concentrations were determined by graphite furnace atomic absorption spectrophotometry (Shimadzu AA-6880, Shimadzu Corporation, Kyoto, Japan). The TF was calculated as:

TF = C_aboveground_/C_root_

where C_aboveground_ is the heavy metal concentration in stems and leaves (mg·kg^−1^), and Cᵣ_oot_ is the heavy metal concentration in roots (mg·kg^−1^).

### 4.4. Determination of Pb, Cu, Zn Subcellular Distribution

The subcellular distribution of Pb, Cu, and Zn was determined according to the method of Honglei Jia [108], with a minor modification wherein the sample weight was increased from 0.1 g to 0.5 g. Briefly, the aboveground plant tissues were homogenized in a pre-cooled extraction buffer containing 250 mmol·L^−1^ sucrose, 50 mmol·L^−1^ Tris-HCl (pH 7.5), and 1.0 mmol·L^−1^ dithiothreitol. The homogenate was centrifuged at 300× *g* for 15 min, and the resulting precipitate was designated as the cell wall fraction. The supernatant was then centrifuged at 2000× *g* for 30 min, yielding a pellet (organelle fraction) and a supernatant (soluble fraction). Heavy metal content in each fraction was quantified following the digestion and analytical procedures described in Section 4.3.

### 4.5. Measurement of Photosynthetic Pigment Content

Fresh leaf samples (0.5 g) were immersed in 10 mL of 95% ethanol in a 20 mL tube. Absorbance was measured at 470 nm, 665 nm, and 649 nm using a spectrophotometer [109] (UV-5200, Shanghai METASH Instruments Co., Ltd., Shanghai, China). The concentrations of chlorophyll a, chlorophyll b, and carotenoids were calculated using the following formulae:

Chlorophyll a (C_a_, mg·L^−1^) = 13.95A_665_ − 6.88A_649_

Chlorophyll b (C_b_, mg·L^−1^) = 24.96A_649_ − 7.32A_665_

Carotenoids (C_car_, mg·L^−1^) = (1000A_470_ − 2.05C_a_ − 114.8C_b_)/245

Total chlorophyll content = C_a_ + C_b_

### 4.6. Determination of Osmoregulatory Substances

The contents of osmoregulatory substances, proline, soluble protein, soluble sugars, and reducing sugars, were measured. Free proline content was determined using the acid-ninhydrin method. Soluble protein content was quantified with Coomassie Brilliant Blue G-250 staining. Soluble sugars were analyzed by the anthrone-sulfuric acid method, and reducing sugars were measured using the 3,5-dinitrosalicylic acid (DNS) method [110].

### 4.7. Oxidative Stress-Related Indicators Measurements

MDA content was determined using the thiobarbituric acid (TBA) method, with absorbance measured at 532 nm and 600 nm. GSH content in *A. argyi* leaves was quantified using a commercial assay kit (A006-1-1, Nanjing Jiancheng Bioengineering Research Institute, Nanjing, China). Non-protein thiol (NPT) content was measured using another assay kit (BC1430, Solarbio Life Sciences, Beijing, China). All reagents, samples, and standards were prepared according to the manufacturer’s instructions, and calculations were performed as specified. PC content in roots was calculated by subtracting GSH content from NPT content [108].

SOD activity was assayed in fresh leaf samples homogenized in 0.1 mol·L^−1^ phosphate buffer (pH 7.0). The homogenate was centrifuged at 15,000× *g* and 4 °C for 30 min. SOD activity was determined according to Alsamadany and Hameed [111], based on the amount of enzyme required to achieve 50% inhibition of nitro blue tetrazolium reduction.

H_2_O_2_ concentration in *A. argyi* leaves was determined using a commercial assay kit (A064-1-1, Nanjing Jiancheng Bioengineering Research Institute, China). Approximately 0.1 g of tissue was homogenized with 1 mL reagent on ice and centrifuged at 8000× *g* (10 min, 4 °C). The complete supernatant was collected for absorbance measurement at 415 nm against a standard. All reagents, samples, and standards were prepared following the manufacturer’s protocol, and calculations were performed as specified.

### 4.8. Statistical Analysis

The mean and standard deviation (SD) were calculated using WPS Office (version 12.1.0). One-way analysis of variance (ANOVA) was performed in R to determine statistical significance, and means were compared using the least significant differences (LSD) test at a significance level of *p* = 0.05. Pearson correlation coefficients were obtained using R (version 4.2.2). Data in bar charts are presented as means, with error bars representing SD. Results are expressed as means ± SD.

## 5. Conclusions

This study provides fundamental insights into the distribution and physiological responses of *A. argyi* to Pb, Cu, and Zn stress, with reference to Chinese national standard values for heavy metals in agricultural soils. The findings indicate that exposure to Pb, Cu, and Zn induced substantial ROS generation in *A. argyi*, resulting in increased levels of antioxidants such as GSH and MDA. Exposure to high concentrations of Cu and Zn impaired the antioxidant system, resulting in decreased antioxidant content. Correlation analysis between growth and physicochemical indicators revealed that Pb, Cu, and Zn stress significantly inhibited photosynthetic pigment production in *A. argyi*. Under Pb treatment, consistent observations in biomass and metal accumulation demonstrated strong tolerance of *A. argyi* to Pb. Subcellular analysis indicated that the majority of Pb was bound to the cell wall, whereas Cu and Zn were further detoxified within soluble cellular components. This differential behavior resulted in predominant root enrichment of Pb, while Cu and Zn exhibited higher translocation rates. These findings demonstrate that *A. argyi* employs multiple effective mechanisms to mitigate heavy metal stress. The species exhibits a strong accumulation capacity and high survival rate under such conditions. Its notable tolerance to Pb, combined with predominant root enrichment, supports its potential use in long-term phytostabilization and vegetation restoration of Pb-contaminated soils, with additional economic value owing to minimal aboveground transfer (though product safety requires further verification). Given the high mobility of Zn, *A. argyi* could be utilized in repeated phytoextraction programs to gradually reduce soil Zn concentrations. For Cu-contaminated soils, further research may focus on the use of amendments to enhance translocation or mitigate accumulation. Overall, this study provides a systematic evaluation of heavy metal accumulation patterns and physiological responses in *A. argyi*, underscoring its promise as a multipurpose phytoremediation crop.

## Data Availability

The original contributions presented in this study are included in the article. Further inquiries can be directed to the corresponding author.

## Abbreviations

HPb	High-Pb
LPb	Low-Pb
MPb	Medium-Pb
LCu	Low-Cu
MCu	Medium-Cu
HCu	High-Cu
LZn	Low-Zn
MZn	Medium-Zn
HZn	High-Zn
